# Smart Polymeric Micelles with Aggregation-Induced Emission and pH-Responsive Fluorescence Color Change Behavior for Bioimaging and Cancer Therapy

**DOI:** 10.3390/ijms26146654

**Published:** 2025-07-11

**Authors:** Zhenrong Liu, Zhe Zong, Xiaoxin Li, Shaoping Sun

**Affiliations:** Department of Pharmaceutical Engineering, School of Chemistry and Material Science, Heilongjiang University, Harbin 150080, China; liuzhenrong521@163.com (Z.L.); zongzhe626@163.com (Z.Z.); lixiaoxin626@163.com (X.L.)

**Keywords:** polymeric micelles, aggregation-induced emission, pH sensitivity, fluorescence discoloration

## Abstract

In this paper, a multifunctional polymer BT-PGA-TPE-HNPE was designed and synthesized by modifying γ-polyglutamic acid (γ-PGA) with biotin, the tetraphenylethylene derivative O-TPE-HNPE and an acid-sensitive imine bond. The polymer was used to fabricate paclitaxel (PTX)-loaded micelles. As expected, the BT-PGA-TPE-HNPE micelles demonstrated strong AIE characteristics, fluorescing yellow under normal conditions and blue in acidic settings. Moreover, the drug was specifically released under acidic conditions. In vitro and in vivo tumor suppression experiments showed that the micelles had enhanced antitumor activity with minimal systemic toxicity. The BT-PGA-TPE-HNPE micelles had wide application prospects in the fields of chemotherapy and bioimaging.

## 1. Introduction

Polymeric micelles have garnered growing interest over the last ten years as cancer drug carriers, owing to their ability to improve the solubility and stability of anticancer agents [1,2]. Generally, polymer micelles are usually amphiphilic polymers self-assembled in an aqueous medium. Their unique core–shell structure enables the micelles to provide drug loading space for hydrophobic drugs. Moreover, micelles with appropriate particle size (20–200 nm) are able to take advantage of the enhanced permeability and retention (EPR) effect, so as to accumulate in tumor tissues [3,4].

Polyglutamic acid (γ-PGA) is a natural non-toxic biopolymer composed of repeating units of D-glutamic acid and L-glutamic acid [5]. Due to the negatively charged glutamic acid residues, γ-PGA exhibits a polyanionic behavior in aqueous solutions, which enables it to possess excellent solubility [6]. Meanwhile, γ-PGA has good biocompatibility and biodegradability. It can be degraded by enzymes in living organisms and release its glutamic acid monomer [7]. Taking γ-PGA as the hydrophilic segment of micelles would effectively enhance the solubility of drugs without causing any toxic side effects.

It was reported that the EPR effect is very limited to enable nanoparticles to accumulate in the tumor. Inspired by the overexpression of receptors on the surface of tumor cells, it is feasible to incorporate specific ligands into micelles, so as to deliver the drug to the target cells through receptor-mediated endocytosis [8,9,10]. Biotin, as an essential cellular micronutrient, is critical for cell function, growth, and development [11]. Using biotin as an active targeting ligand, the nanoparticles would be able to deliver drugs specifically to cancer cells, thus minimizing toxicity to healthy cells [12].

As is well known, insufficient drug release at the target site leads to low drug bioavailability and even drug resistance [13,14]. The design of drug delivery systems with environmentally sensitive release capabilities holds broad promise. Considering the acidic microenvironment of tumors, the introduction of an acid cleavable bond would render the micelles a tumor-specific drug release behavior. Generally, imine bonds are used for the fabrication of pH-sensitive nanoparticles [15,16,17]. In an acidic tumor environment, the nitrogen atom is protonated, which leads to the cleavage of the imine bond and the precise release of antitumor drugs. It is clear that the combination of active targeting and stimulus-responsive drug release would greatly enhance the drug concentration at the tumor site [18]. However, an ideal antitumor drug delivery system should also be able to track the fate of particles in the body, which requires bioimaging capabilities [19].

Fortunately, the discovery of aggregation-induced luminescence (AIE) offers this possibility. AIE is a unique photophysical phenomenon first observed by Tang et al. in 2001 [20]. Fluorescent materials with the AIE feature emit weak or even no luminescence in a dispersed state, but their luminescence intensity increases significantly in an aggregated or solid state [21,22]. This phenomenon is extremely different from the aggregation-caused quenching (ACQ) of traditional fluorescent molecules and provides a feasible approach for the bioimaging of micelles [23]. As is well known, tetraphenylethylene (TPE) is a typical group with the AIE effect, which has attracted great attention [24]. TPE has a propeller-like structure, consisting of four peripheral phenyl groups (aromatic rotors) bonded to a central double bond [25]. TPE is not luminescent in dilute solutions because the relatively free rotation of the phenyl ring allows energy to be consumed mainly through non-radiative decay. In contrast, in the aggregated state, TPE emits strong fluorescence due to the restriction of its intramolecular rotation (RIR) [26]. In view of good designability and easy modification, TPE could be endowed with the capability of altering its fluorescence color in different environments through the introduction of sensitive bonds and modifications. Specifically, modifying TPE with 2-hydroxy-1-naphthaldehyde via the Schiff base reaction would allow for the extension of the conjugation plane, and an excited state intramolecular proton transfer (ESIPT)-based compound with a hydrogen donor–acceptor structure (O−H and C=N) would be formed [27]. This polymer might exhibit redshifted emissions. Moreover, the imine bond generated would enable the system to change its color in acidic environments, providing the capability for real-time drug release monitoring [28,29].

Inspired by the above-mentioned information, in this study, a novel polymer BT-PGA-TPE-HNPE was designed and synthesized. This polymer was prepared by modifying γ-PGA with a TPE derivative and active targeting ligand biotin, with an acid-sensitive imine bond in the molecule. Due to its amphiphilicity, the hydrophobic drug paclitaxel (PTX) could be encapsulated to form drug-loaded BT-PGA-TPE-HNPE micelles. It was speculated that the micelles would possess desirable AIE properties. Moreover, in the acidic tumor microenvironment, the fluorescence color would be changed upon the breakage of the imine bond and the drug would be specifically released there, thus realizing the real-time monitor of drug release and enhanced drug concentration in the tumor. The fabrication of the PTX-loaded BT-PGA-TPE-HNPE micelles and their antitumor efficacy and bioimaging effect were investigated in depth.

## 2. Results and Discussion

### 2.1. Synthesis Route of BT-PGA-TPE-HNPE

**Scheme 1:**
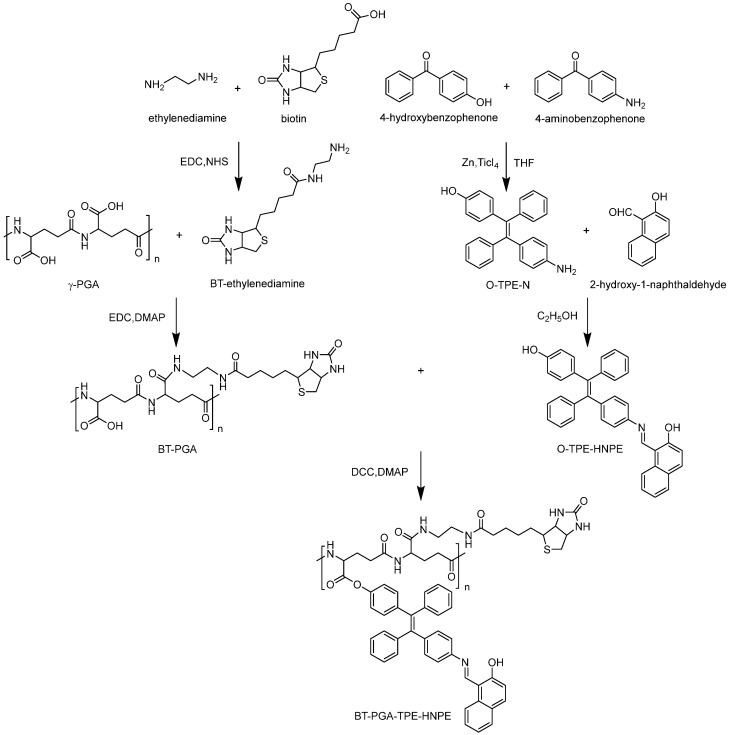
Synthesis route of BT-PGA-TPE-HNPE.

The synthesis process of BT-PGA-TPE-HNPE was briefly summarized in Scheme 1. On the one hand, O-TPE-HNPE was prepared using a two-step reaction. Firstly, 4-aminobenzophenone reacted with 4-hydroxybenzophenone via the McMurray reaction to produce O-TPE-N. Then, a Schiff base reaction was performed between O-TPE-N and 2-hydroxy-1-naphthaldehyde, and O-TPE-HNPE was obtained with a pH-sensitive imine bond in the structure. On the other hand, using ethylenediamine as the connecting arm, biotin was conjugated with γ-PGA through amide reactions, leading to the formation of BT-PGA. At last, the carboxyl groups of BT-PGA were linked with the hydroxyl groups of O-TPE-HNPE via the formation of ester bonds, and BT-PGA-TPE-HNPE was obtained.

### 2.2. Analysis and Characterization of BT-PGA-TPE-HNPE

#### 2.2.1. ^1^H NMR Analysis

**Figure 1:**
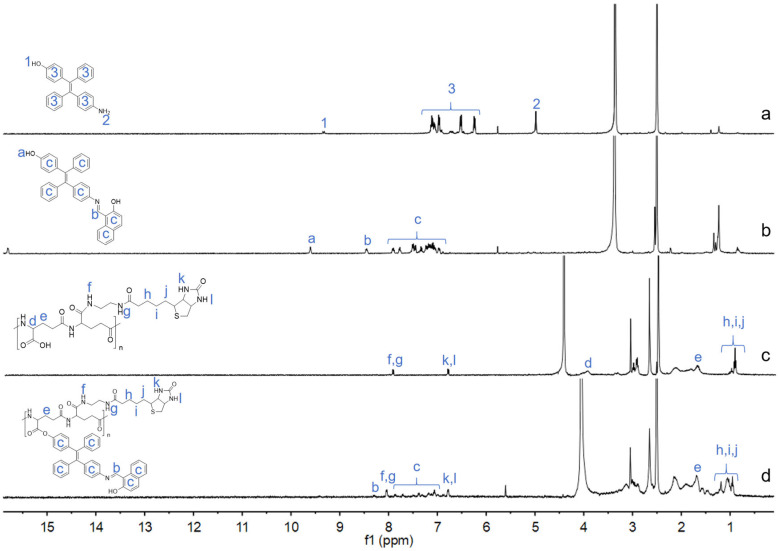
^1^H NMR spectra of (**a**) O-TPE-N, (**b**) O-TPE-HNPE, (**c**) BT-PGA and (**d**) BT-PGA-TPE-HNPE.

To confirm the formation of BT-PGA-TPE-HNPE, a ^1^H NMR analysis was conducted. As shown in Figure 1, in the spectrum of O-TPE-N, signals at 9.33 ppm (H-1) and 4.98 ppm (H-2) were attributed to the phenol hydroxyl protons and the amino protons, respectively. For O-TPE-HNPE, the signal assigned to the amino protons at 4.98 ppm (H-2) disappeared. New signals observed at 9.59 ppm (H-a) and 8.45 ppm (H-b) were ascribed to the phenol hydroxyl protons on tetraphenylene and the imine protons, respectively, which demonstrated the successful synthesis of O-TPE-HNPE. In the spectrum of BT-PGA, signals located at 1.69–1.91 ppm (H-e) and 4.02 ppm (H-d) belonged to the methylene protons and methane protons of γ-PGA, respectively. Signals attributed to the protons in the structure of biotin were presented at 6.82 ppm (H-k, l) and 0.93–1.00 ppm (H-h, i, j). New signals at 7.95–7.93 ppm (H-f, g) were assigned to the amide protons, which were generated via the reaction of ethylenediamine with the carboxyl groups of γ-PGA and biotin. For BT-PGA-TPE-HNPE, the signal assigned to the imine protons of O-TPE-HNPE was still observed at 8.03 ppm (H-b), while the signal ascribed to the phenolic hydroxyl protons of O-TPE-HNPE (H-a) disappeared completely. Moreover, the signal attributed to the amide protons was shifted to 8.05–8.03 ppm (H-f, g). All these pieces of evidence demonstrated the successful formation of BT-PGA-TPE-HNPE. From the integration unit ratio, the substitution degree of biotin and O-TPE-HNPE for γ-PGA was about 15.3% and 6.2%, respectively.

#### 2.2.2. FTIR Analysis

**Figure 2:**
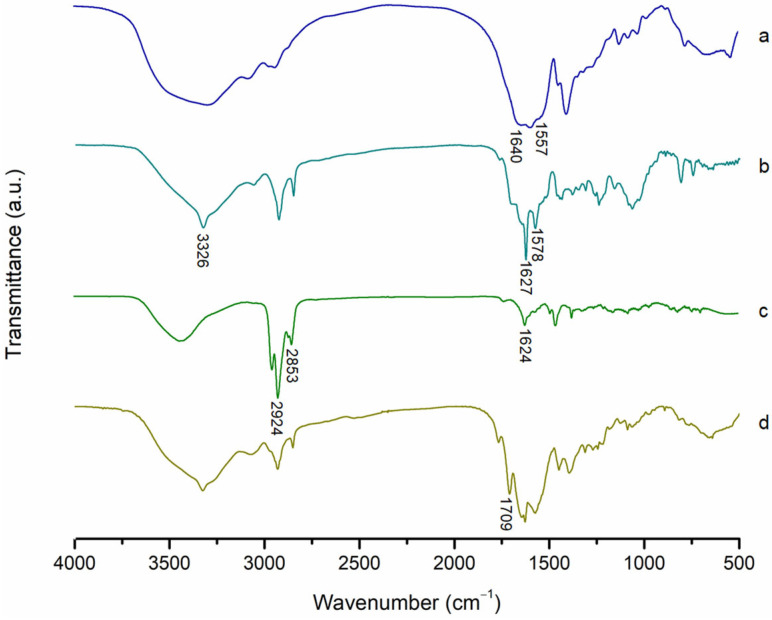
FTIR spectra of (**a**) γ-PGA, (**b**) BT-PGA, (**c**) O-TPE-HNPE and (**d**) BT-PGA-TPE-HNPE.

FTIR analysis was also performed to further confirm the successful synthesis of the polymer. As shown in Figure 2, in the spectrum of γ-PGA, strong absorption bands at 1640 cm^−1^ and 1557 cm^−1^ were ascribed to the stretching vibration of the amide I band and amide II band, respectively. In comparison, for BT-PGA, the characteristic bands of the amide bond were shifted to 1627 cm^−1^ and 1578 cm^−1^, respectively. Moreover, a new absorption band attributed to the stretching vibration of the N−H bond was observed at 3326 cm^−1^, suggesting the connection of biotin to γ-PGA. In the spectrum of O-TPE-HNPE, bands at 2924 cm^−1^ and 2853 cm^−1^ were attributed to the stretching vibration of =CH− and −CH− on the benzene ring. The characteristic band at 1624 cm^−1^ was due to the stretching vibration of −C=N, which indicated the connection of the amino group of O-TPE-N with the aldehyde group of 2-hydroxy-1-naphthaldehyde. For BT-PGA-TPE-HNPE, the newly generated absorption band at 1709 cm^−1^ was assigned to the C=O stretching vibration of the ester bond, which was formed between the alcohol hydroxyl groups of O-TPE-HNPE and the carboxyl groups of BT-PGA. These results indicated that BT-PGA-TPE-HNPE was successfully synthesized.

#### 2.2.3. Optical Properties Investigation

**Figure 3:**
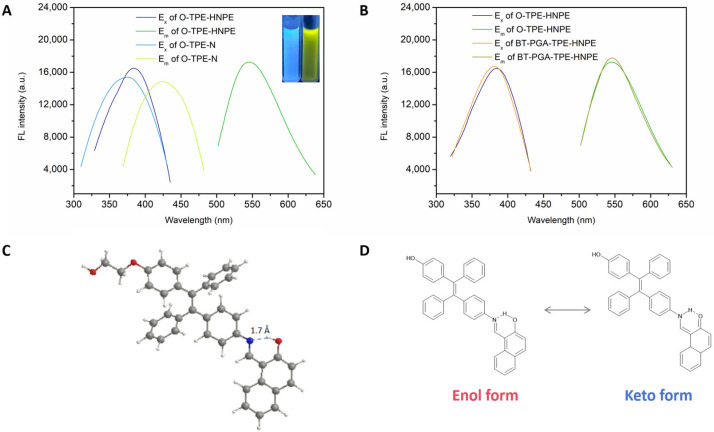
(**A**) Fluorescence excitation (E_x_) and emission (E_m_) spectra of O-TPE-N (E_x_ = 375 nm, E_m_ = 425 nm) and O-TPE-HNPE (E_x_ = 384 nm, E_m_ = 546 nm) in water. Inset: Images of O-TPE-N and O-TPE-HNPE solutions taken under a 365 nm UV lamp. (**B**) Fluorescence excitation and emission spectra of BT-PGA-TPE-HNPE (E_x_ = 381 nm, E_m_ = 546 nm) in water. (**C**) 3D molecular structure diagram of O-TPE-HNPE. (Red represents oxygen atoms, blue represents nitrogen atoms, gray represents carbon atoms, and white represents hydrogen atoms.) (**D**) Transformation of ketone and enol forms of O-TPE-HNPE.

As BT-PGA-TPE-HNPE was a Schiff-base derivative of TPE, the polymer was expected to exhibit excellent pH-sensitive AIE characteristics. The fluorescence behaviors of polymers were investigated. As shown in Figure 3A, for O-TPE-N, the maximum excitation wavelength was 375 nm, and the maximum emission wavelength was 425 nm. From Figure 3B, it was observed that the values for BT-PGA-TPE-HNPE were 381 nm and 546 nm, respectively. Moreover, the excitation and emission spectra of BT-PGA-TPE-HNPE were almost identical to those of O-TPE-HNPE (E_x_ = 384 nm, E_m_ = 546 nm). For O-TPE-HNPE, the excitation band was completely separated from the emission band, with the Stokes shift as high as 162 nm. It was speculated that the large Stokes shift might be due to the ESIPT effect of O-TPE-HNPE. To obtain a deeper understanding, the geometric structure of O-TPE-HNPE was analyzed through theoretical calculations. As presented in Figure 3C, a strong intramolecular hydrogen bond (CH=N⋯HO) could be formed between the active phenolic hydroxyl group (proton donor) and the imine nitrogen atom (proton acceptor) with a minimum bond length of 1.7 Å. As illustrated in Figure 3D, the proton could transfer from –OH to –CH=N through the intramolecular hydrogen bond, leading to a tautomer between ketone and enol forms in the excited state. When the enol form in the ground state was photoexcited, the proton was easily transferred from −OH to the neighboring nitrogen atom, forming a ketone structure, thus reducing the energy. Moreover, due to the formation of the intramolecular hydrogen bond, an additional six-membered ring structure was formed. With a relatively planar conformation, O-TPE-HNPE would emit strong fluorescence in the aggregated state [30]. The fluorescence quantum yield of BT-PGA-TPE-HNPE was estimated to be 26.6%.

**Figure 4:**
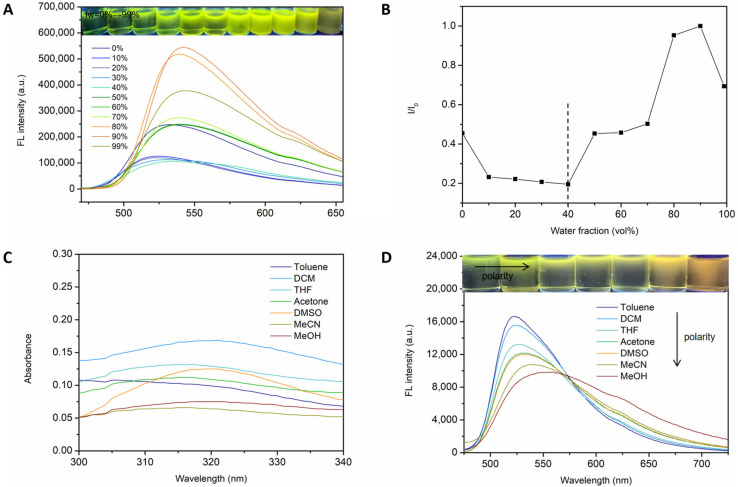
(**A**) Fluorescence emission spectra of O-TPE-HNPE in THF/water mixtures with different f_w_. Inset: Images of O-TPE-HNPE solution with f_w_ from 0 to 99% taken under a 365 nm UV lamp. (**B**) Plot of relative FL intensity (I/I_0_) versus f_w_. I_0_ was the maximum FL intensity of O-TPE-HNPE in the solvent, and I was the FL intensity of each subsequent determination. (**C**) UV-Vis spectra of O-TPE-HNPE in different solvents. (**D**) Fluorescence spectra of O-TPE-HNPE in different solvents. Inset: Images of O-TPE-HNPE in different solvents taken under a 365 nm UV lamp.

To investigate the AIE characteristics of O-TPE-HNPE, the emission spectra in a series of THF/water mixtures were determined using THF as the good solvent and water as the bad solvent. As shown in Figure 4A, with the increase in f_w_, the fluorescence intensity was gradually enhanced. This could be due to the decrease in the solubility of O-TPE-HNPE, which led to the aggregation of the molecules. As a result, the intramolecular motions were restricted, and the emission was significantly enhanced. However, when f_w_ reached 99%, the fluorescence intensity decreased slightly, which could be explained by the precipitation of the polymer. To conduct a more in-depth investigation, the change in fluorescence intensity with f_w_ was determined. As presented in Figure 4B, when f_w_ increased from 0% to 40%, the emission band was redshifted and the fluorescence intensity decreased, which could be due to the change in the polarity of the solvent [31].

To prove this inference, the optical behavior of O-TPE-HNPE in different solvents was measured. From Figure 4C, it was observed that the solvent had little effect on the absorption spectra of O-TPE-HNPE. In contrast, as illustrated in Figure 4D, the fluorescence emission spectra of O-TPE-HNPE exhibited an obvious solvent effect. To be more specific, in low-polar solvent toluene, the fluorescence intensity of the polymer was extremely high, with its maximum emission wavelength at 523 nm. As the solvent polarity increased, the fluorescence intensity decreased, and its emission maxima was redshifted. This phenomenon could be explained by a twisted intramolecular charge transfer (TICT). More specifically, the excited state non-radiative decay process involving TICT from the donor tetrastyrene group to the acceptor naphthalene ring became easier in polar solvents, leading to a decrease in fluorescence intensity and a redshift in emission wavelength [32,33].

**Figure 5:**
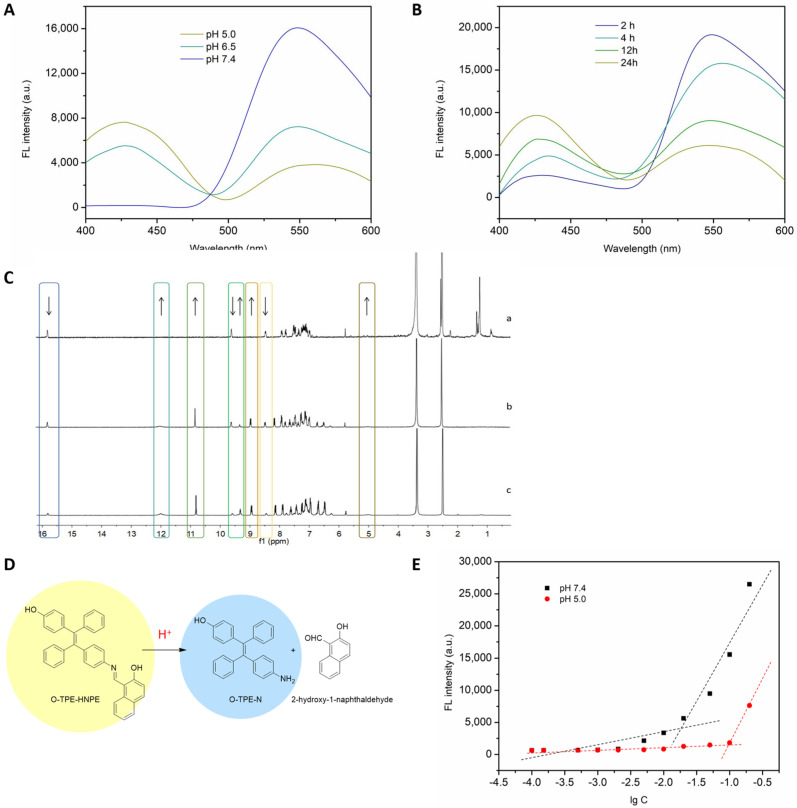
(**A**) Fluorescence spectra of O-TPE-HNPE at different pH values. (**B**) Fluorescence spectra of O-TPE-HNPE after incubation in PBS 6.5 for different durations. (**C**) ^1^H NMR spectra of O-TPE-HNPE after incubation for 4 h at: (a) pH 7.4, (b) pH 6.5, and (c) pH 5.0. (The direction of the arrow indicates the change in the intensity of the characteristic peak.) (**D**) Mechanism of pH-sensitive fluorescent color change. (**E**) Fluorescence intensity as a function of logarithm of copolymer concentration in different pH conditions.

The fluorescence behavior of O-TPE-HNPE at different pH was measured to determine whether the polymer possessed pH-sensitive fluorescence. As shown in Figure 5A, as pH decreased, the fluorescence intensity at 546 nm corresponding to O-TPE-HNPE decreased, while the fluorescence intensity at 425 nm corresponding to O-TPE-N increased. As presented in Figure 5B, the same trend was also observed with the increase in incubation time. This phenomenon could be explained by the presence of the acid-sensitive imine bond in the polymer. At low pH, the imine bond was broken and the polymer decomposed to form O-TPE-N and 2-hydroxy-1-naphthaldehyde, resulting in the change of fluorescence behavior. With its pH-sensitive fluorescence color change property, BT-PGA-TPE-HNPE might have a broad application prospect in the field of bioimaging.

To obtain a deeper insight into the pH-sensitivity of O-TPE-HNPE, the structural changes in the polymer after incubation in acidic environments were determined at a monomolecular level using ^1^H NMR analysis. As presented in Figure 5C, as the acidity of the environment increased, the intensity of the signal attributed to the phenolic hydroxyl groups of O-TPE-HNPE at 9.59 ppm decreased, while the intensity of the signal assigned to the phenolic hydroxyl group of O-TPE-N at 9.33 ppm increased. Moreover, the signal at 8.45 ppm ascribed to the imine bonds in O-TPE-HNPE exhibited a decreased intensity, while the signal assigned to the amino protons of O-TPE-N appeared at 5.00 ppm, and its intensity was enhanced. These pieces of evidence indicated the conversion of O-TPE-HNPE to O-TPE-N upon acid treatment. In addition, the intensity of the signal corresponding to the phenolic hydroxyl protons on the naphthalene ring of O-TPE-HNPE at 15.81 ppm exhibited an obvious decrease. New signals were observed at 12.04 ppm, 10.82 ppm, and 8.94 ppm, which were ascribed to the phenol hydroxyl protons, aldehyde protons, and C−H protons of 2-hydroxy-1-naphthaldehyde, respectively. Furthermore, their intensities increased with the decrease in pH. These differences implied the release of 2-hydroxy-1-naphthaldehyde. It could be deduced that O-TPE-N and 2-hydroxy-1-naphthaldehyde were the main products of the acid-catalyzed hydrolysis of O-TPE-HNPE (Figure 5D).

#### 2.2.4. CMC Determination

The CMC values were measured at two pH values, 7.4 and 5.2. Given that BT-PGA-TPE-HNPE exhibited a perfect AIE performance, the polymer itself could be used as a fluorescent probe for CMC measurement. As presented in Figure 5E, the CMC value of BT-PGA-TPE-HNPE was calculated to be 0.0186 mg/mL following incubation at pH 7.4, which was substantially lower than the CMC values of low-molecular-weight surfactants. This low CMC value indicated that the BT-PGA-TPE-HNPE micelles possessed high stability, such that their structure was not prone to alteration after entering the bloodstream. Under acidic pH conditions, a notable increase in the copolymer’s CMC value was observed, suggesting that the cleavage of imine bonds under acidic conditions made it more difficult for the copolymer to form micelles in the BT-PGA-TPE-HNPE micelle system.

### 2.3. Characterization of BT-PGA-TPE-HNPE Micelles

The hydrodynamic diameter of BT-PGA-TPE-HNPE micelles, crucial for assessing their potential for intravenous use, was measured using DLS. The mean particle size of the optimized blank micelles was 93 ± 1.7 nm (PDI = 0.173), while the mean particle size of the PTX-loaded micelles was 109 ± 3.9 nm (PDI = 0.292). The relatively small particle size would facilitate the passive accumulation of the micelles in tumor tissues through the EPR effect. From a zeta potential measurement, it was noted that the BT-PGA-TPE-HNPE micelles were negatively charged, with a value of −28.13 ± 1.3 mV. The high zeta potential indicated a strong repulsion between particles and the high stability of the micelles. In addition, the negative charge would protect the micelles from clearance by the mononuclear phagocyte system, thus prolonging their circulation time and enhancing the EPR effect [34].

For BT-PGA-TPE-HNPE micelles, the hydrophobic O-TPE-HNPE not only provided pH-sensitive imine bonds but also served as a hydrophobic core to load hydrophobic anticancer drugs. HPLC analysis showed that the PTX-loaded micelles possessed a high drug-carrying capacity and high encapsulation efficiency, with values of 15.0% and 90.1%, respectively.

**Figure 6:**
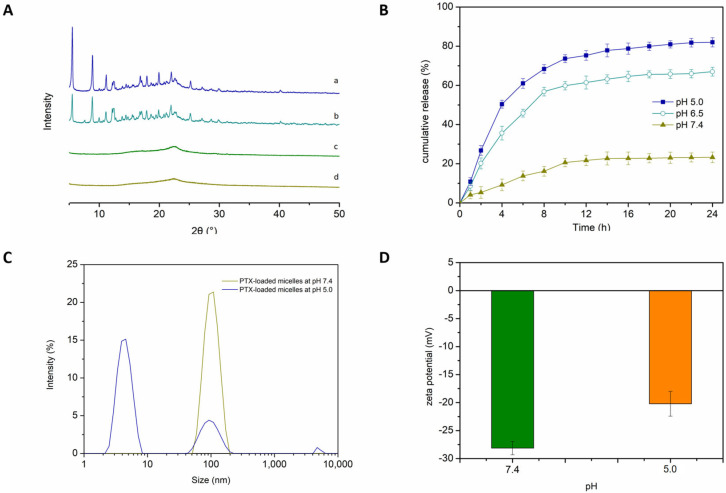
(**A**) XRD patterns of (a) PTX, (b) the physical mixture of PTX and blank micelles, (c) blank BT-PGA-TPE-HNPE micelles, and (d) PTX-loaded BT-PGA-TPE-HNPE micelles. (**B**) Drug release profiles of PTX-loaded BT-PGA-TPE-HNPE micelles in PBS with different pH. (**C**) Particle size distribution profiles of micelles after incubation with PBS 7.4 and PBS 5.0. (**D**) Zeta potential distribution of micelles after incubation with PBS 7.4 and PBS 5.0.

The presence state of PTX in BT-PGA-TPE-HNPE micelles was analyzed using an XRD analysis. As shown in Figure 6A, the physical mixture exhibited a superposition of the characteristic peaks of PTX and blank micelles. Unlike blank micelles, the PTX-loaded micelles did not exhibit distinct peaks corresponding to PTX in their spectra. This suggests that the drug was effectively encapsulated within the micelles, likely in an amorphous or molecular form, thus confirming successful preparation.

### 2.4. pH-Responsive Drug Release Behavior

The incorporation of an acid-labile imine bond into the polymer was expected to render the micelles an acid-triggered drug release behavior. In order to evaluate the effect of pH on drug release, PBS solutions with different pH were used as the release media. As shown in Figure 6B, the drug release behavior was significantly affected by the pH of the medium, that is, drug release increased with the decrease in pH. At a pH of 7.4, a slow drug release was observed, with only 25.2% of PTX released within 24 h. In comparison, at a pH of 6.5, the release of PTX was much faster, with 68.9% of the drug released within 24 h. When the pH of the medium was as low as 5.0, the drug release was further facilitated and 81.9% of PTX was released within 24 h. The reason for this phenomenon was the presence of imine bonds in the micelles. To be specific, imine bonds are stable at physiological pH. However, they would break when in acidic environments, thereby promoting the disassembly of micelles and facilitating the release of the drug. These results suggested that the developed PTX-loaded micelles would maintain structural integrity in blood circulation but release drugs efficiently in tumors. The selective release of drugs in tumors would improve the efficacy of the drugs and reduce toxicity.

The DLS results further corroborate the pH-responsive property of the micelles. As shown in Figure 6C, upon incubation with PBS 5.0 for 24 h, the size distribution transformed from a single peak to multiple peaks, evidencing the disintegration of the micelles. This observation implied that BT-PGA-TPE-HNPE micelles preserved their structural integrity during blood circulation while undergoing rapid decomposition to release drugs in acidic tumor tissues. Furthermore, the variation in zeta potential was assessed (Figure 6D). Following 24 h of incubation in PBS 5.0, the sample potential exhibited a decrease, which was attributed to the cleavage of imine bonds. The exposure of amino groups reduced the original negative zeta potential value, further verifying that BT-PGA-TPE-HNPE micelles enabled specific drug release in tumor microenvironments.

### 2.5. Cellular Imaging and Cellular Uptake Behavior

**Figure 7:**
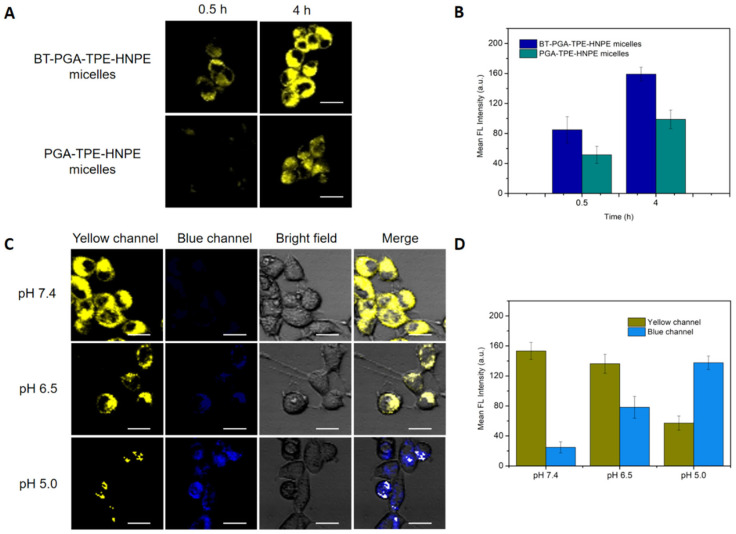
(**A**) Fluorescence images of 4T1 cells after incubation with BT-PGA-TPE-HNPE micelles or PGA-TPE-HNPE micelles for different durations. Scale bar: 20 μm. (**B**) Mean fluorescence intensity of the yellow channel measured by Image J software after incubation with BT-PGA-TPE-HNPE micelles or PGA-TPE-HNPE micelles for different durations. (**C**) Fluorescence images of 4T1 cells after incubation with BT-PGA-TPE-HNPE micelles in different pH conditions. Scale bar: 20 μm. (**D**) Mean fluorescence intensity of different fluorescence signals measured by Image J software after incubation with BT-PGA-TPE-HNPE micelles under different pH values.

Effective uptake by tumor cells was an indicator of the good therapeutic effect of the micelles. Considering their excellent AIE property, the cellular internalization behavior of BT-PGA-TPE-HNPE micelles was directly observed by CLSM, so as to evaluate the trace ability and uptake behavior of the micelles. As shown in Figure 7A, yellow fluorescence was clearly visible in the cytoplasm of cells after incubation with BT-PGA-TPE-HNPE micelles for 0.5 h. It was indicated that the micelles had a strong cell imaging ability, and they were easily internalized by tumor cells. As the incubation time extended to 4 h, the fluorescence intensity was increased. In comparison, under the same conditions, the PGA-TPE-HNPE micelles exhibited much weaker fluorescence. The fluorescence intensity measured by the Image J software (https://ij.imjoy.io/, accessed on 18 December 2024) is illustrated in Figure 7B. It was deduced that compared with PGA-TPE-HNPE micelles, more BT-PGA-TPE-HNPE micelles were endocytosed by 4T1 cells and the micelles exhibited remarkable bioimaging ability. A possible explanation was that due to the over-expression of biotin receptors on tumor cells, the modification by biotin would facilitate the endocytosis of micelles via receptor-mediated internalization, and the excellent AIE feature made the micelles self-tracing. In addition, the modification of γ-PGA improved the biocompatibility of the hydrophobic AIE molecule. Meanwhile, when the polymeric micelles aggregated at the tumor site, the presence of hydrophilic macromolecules further restricted the internal rotation of the fluorescent molecules, thereby improving fluorescence performance. This enhancement was crucial for monitoring the micelles’ behavior in vivo.

The fluorescence color behavior of BT-PGA-TPE-HNPE micelles under different pH conditions was also detected after incubation for 4 h. As shown in Figure 7C, bright yellow fluorescence was observed in the cytoplasm at a pH of 7.4. At a pH of 6.5, both yellow and blue fluorescence were detected in the cytoplasm. While at a pH of 5.0, much brighter blue fluorescence and weaker yellow fluorescence were observed. The mean fluorescence intensity value of each image is presented in Figure 7D. These results demonstrated that with the decrease in pH, the intensity of the yellow fluorescence decreased, while the blue fluorescence intensity increased, which renders the BT-PGA-TPE-HNPE micelles an excellent pH-sensitive color changeable property for the real-time monitor of drug release.

### 2.6. In Vitro Cytotoxicity Assay

**Figure 8:**
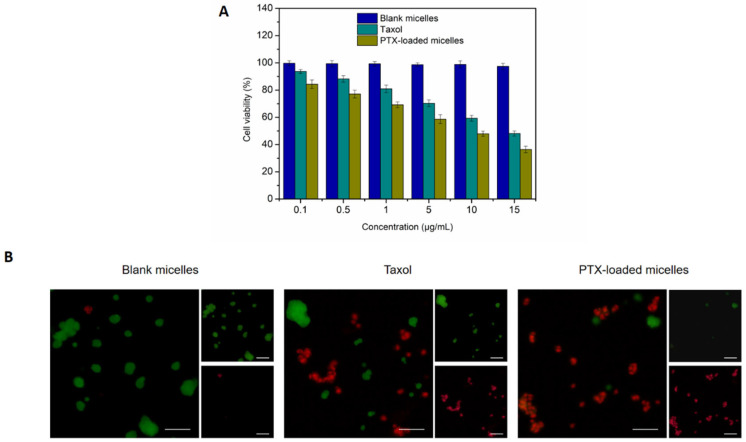
(**A**) Cytotoxicity of PTX-loaded BT-PGA-TPE-HNPE micelles on 4T1 cells. (**B**) Live/dead staining images of 4T1 cells after various treatments. Scale bar: 40 μm.

The MTT method was used to evaluate the cytotoxicity of PTX-loaded BT-PGA-TPE-HNPE micelles. As shown in Figure 8A, more than 90% of the cells remained alive after incubation with different concentrations of blank micelles. This result implied that the blank micelles had no significant cytotoxicity and the polymer exhibited good biocompatibility. More excitingly, for micelles loaded with PTX, they showed a much stronger inhibition effect on cell growth than Taxol. Their superior cytotoxicity could be due to the enhanced endocytosis of the micelles and the presence of imine bonds in the molecule. To be specific, more micelles could be internalized by 4T1 cells upon modification by biotin. Once the micelles enter the cells, the acidic environment would trigger the imine bonds to break, thus leading to the quick release of the drug. It could be inferred that BT-PGA-TPE-HNPE micelles have the potential to enhance antitumor activity and reduce the toxic side effects of PTX in vivo [35].

In addition, live/dead cell staining was also carried out to further investigate the killing effect of PTX-loaded BT-PGA-TPE-HNPE micelles on cancer cells. The green fluorescent Calcein AM could stain live cells, while the red fluorescent PI dye could only enter and stain dead cells. As shown in Figure 8B, strong green fluorescence was observed in the blank micelles group. For the PTX formulation groups, a larger red fluorescence area was seen in the PTX-loaded micelles group than the Taxol group, indicating the enhanced killing effect of the drug-loaded micelles. It could be deduced that the PTX-loaded BT-PGA-TPE-HNPE micelles exhibited superior toxicity on tumor cells [36].

### 2.7. In Vivo Antitumor Efficacy Assessment

**Figure 9:**
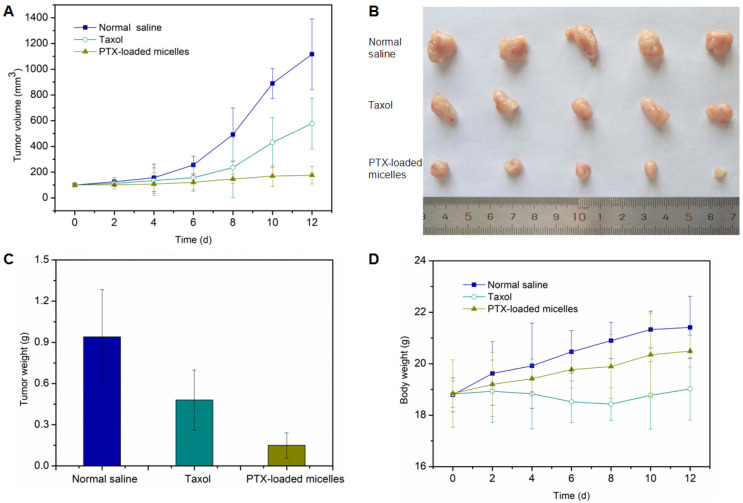
(**A**) Tumor growth curves of mice in different treatment groups. (**B**) Photos of tumors excised from different treatment groups. (**C**) Tumor weight of mice after different treatments. (**D**) Body weight changes in mice in different treatment groups.

The antitumor activity of the PTX-loaded BT-PGA-TPE-HNPE micelles was further evaluated via a tumor growth inhibition study in 4T1 tumor-bearing mice. As shown in Figure 9A, tumors grew rapidly in the saline-treated group, while the Taxol and the PTX-loaded BT-PGA-TPE-HNPE micelles exhibited an evident inhibitory effect on tumor growth. The same trend could also be noted from Figure 9B,C, i.e., the tumors of the mice in the treated groups were much smaller than those in the normal saline group. Furthermore, the micelles-treated group exhibited the smallest tumor sizes, indicating their superior tumor inhibition effect. The design of the BT-PGA-TPE-HNPE micelles played an important role for the superior antitumor efficacy of PTX. Specifically, the BT-PGA-TPE-HNPE micelles could accumulate in tumors through the EPR effect due to their nano-size of about 100 nm. The introduction of the active targeting ligand biotin facilitated the effective entry of micelles into tumor cells. Meanwhile, the negative charge prevented the micelles from being cleared by the mononuclear phagocyte system and increased their circulation time in the blood so as to improve their chances of uptake via the EPR effect. In tumor tissues, the acidic environment triggered the breakage of imine bonds in the micelles, thus promoting the drug release.

In addition, to examine the systemic toxicity of the BT-PGA-TPE-HNPE drug-carrying micelles, changes in body weights of the mice were recorded. As shown in Figure 9D, similar to the saline-treated group, there was no weight loss in the mice treated with PTX-loaded BT-PGA-TPE-HNPE micelles, implying that the micelles were highly biocompatible and did not harm the mice. The BT-PGA-TPE-HNPE micelles were promising carriers for cancer therapy.

Meanwhile, Table 1 presents a detailed comparison between BT-PGA-TPE-HNPE micelles and CD-CS-Bio-TPAA micelles (CCBT), which were prepared in our previous study [37]. As shown in Table 1, BT-PGA-TPE-HNPE exhibited several distinct advantages. Compared with CCBT, BT-PGA-TPE-HNPE could be prepared via a simpler synthesis route, and the formed micellar structure was more stable (with a much lower CMC value). Its drug loading capacity and encapsulation efficiency were also higher than those of CCBT. Moreover, BT-PGA-TPE-HNPE micelles exhibited a higher cumulative drug release rate within 24 h at a pH of 5.0, indicating the superior pH-responsive behavior of BT-PGA-TPE-HNPE.

## 3. Materials and Methods

### 3.1. Materials

γ-Polyglutamic acid (γ-PGA) was purchased from Meiyi Biotechnology Co., Guangzhou, China. 4-Hydroxybenzophenone and 4-aminobenzophenone were purchased from Meryer Chemical Co., Shanghai, China. 2-Hydroxy-1-naphthaldehyde, biotin, ethylenediamine, N-hydroxysuccinimide (NHS), 1,3-dicyclohexylcarbodiimide (DCC), 1-ethyl-(3-dimethylaminopropyl) carbodiimide (EDC), 3-(4,5-dimethylthiazol-2-yl)-2,5-diphenyltetrazolium bromide (MTT), and 4-dimethylaminopyridine (DMAP) were purchased from Aladdin Industry Co., Shanghai, China. Paclitaxel (PTX, purity of 99.9%) was provided by Natural Field Biotechnology Co., Xi’an, China. All reagents required for cell culture including Roswell Park Memorial Institute (RPMI) 1640 medium, fetal bovine serum (FBS) and a penicillin–streptomycin mixture were purchased from Solarbio Science & Technology Co., Beijing, China. All remaining chemicals and reagents were of analytical or HPLC grade and obtained from commercial suppliers.

### 3.2. Cells and Animals

4T1 cells (mouse breast cancer cells) were obtained from Harbin Medical University, Harbin, China. Cells were cultured in RPMI 1640 medium containing 100 μg/mL of penicillin, 100 μg/mL of streptomycin, and 10% FBS. Specific pathogen-free female BALB/c mice (18–20 g) were provided by the Laboratory Animal Center of Harbin Medical University, Harbin, China. The animals were housed under a constant temperature of 25 °C and humidity level of 50–60% with natural light illumination. All animal experiments were conducted in accordance with the ARRIVE guidelines after the approval of the Animal Ethics Committee of Heilongjiang University (No. 20230220005).

### 3.3. Synthesis of BT-PGA-TPE-HNPE

#### 3.3.1. Synthesis of O-TPE-HNPE

Zinc powder (0.5 g) and tetrahydrofuran (THF, 20 mL) were mixed via stirring in an ice-water bath. After the addition of titanium tetrachloride (TiCl_4_, 1.5 mL), the mixture was heated to 40 °C and stirred for 30 min. Then pyridine (1.5 mL) was added, and stirring was continued for another 30 min. Afterwards, 4-aminobenzophenone (0.61 g) and 4-hydroxybenzophenone (0.51 g) in THF (30 mL) solution was added. The reaction was allowed to proceed for 12 h. At the predetermined time point, 10% anhydrous potassium carbonate aqueous solution (100 mL) was added to quench the reaction. The resultant was extracted with dichloromethane (DCM) five times. The collected organic layer was concentrated and purified on a silica gel column using a mixture of petroleum ether/ethyl acetate (5:1, *v*/*v*) as the eluent. The pure TPE derivative of O-TPE-N was obtained as a brown solid.

Then O-TPE-N (0.2 g) and 2-hydroxy-1-naphthaldehyde (0.08 g) were dissolved in anhydrous ethanol (8 mL), and the mixture was refluxed at 80 °C for 6 h. The resultant was precipitated, washed three times with anhydrous ethanol, and filtered under reduced pressure to obtain the pure O-TPE-HNPE as an orange solid.

#### 3.3.2. Synthesis of BT-PGA

Biotin (BT, 100 mg), EDC (63 mg) and NHS (74 mg) were dissolved in N,N-dimethylformamide (DMF, 5 mL). The mixture was stirred at 40 °C for 24 h to activate the carboxyl groups of biotin. Then excess ethylenediamine was added. The reaction was proceeded at 50 °C for 24 h. After filtration, a white solid of biotinylated ethylenediamine (BT-ethylenediamine) was obtained.

On the other hand, γ-PGA in a distilled water solution (700 mg in 10 mL) was mixed with DMAP (661 mg) and EDC (623 mg). The reaction was carried out at 45 °C for 48 h to activate the carboxyl groups of γ-PGA. Then, the BT-ethylenediamine in dimethyl sulfoxide (DMSO) solution (155 mg in 5 mL) was added, and the reaction was carried out at 45 °C for 48 h. Following 24 h of dialysis against distilled water (MWCO of 7 k Da) and subsequent freeze-drying, biotinylated γ-PGA (BT-PGA) powder was produced.

#### 3.3.3. Synthesis of BT-PGA-TPE-HNPE

The polymer BT-PGA-TPE-HNPE was prepared by conjugating BT-PGA with O-TPE-HNPE. Typically, BT-PGA (580 mg), DCC (639 mg), and DMAP (503 mg) were dissolved in a mixture of water and DMSO (5:1, *v*/*v*). The reaction was conducted via stirring at 45 °C for 24 h. Then O-TPE-HNPE (213 mg) was added, and stirring was continued for a further 24 h. The resultant was dialyzed with distilled water (MWCO of 14 k Da). The product BT-PGA-TPE-HNPE was collected by lyophilization.

### 3.4. Characterization of BT-PGA-TPE-HNPE

#### 3.4.1. Confirmation of Polymer Formation

The structure of BT-PGA-TPE-HNPE was characterized via FTIR analysis (Tensor II, Bruker, Switzerland) in the range of 500–4000 cm^−1^ using a potassium bromide compression method [38]. In addition, the polymer was dissolved in a deuterated solvent and analyzed on a Bruker Avance spectrometer (AV-400, Bruker, Karlsruhe, Germany) at 400 MHz to obtain a ^1^H NMR spectra.

#### 3.4.2. Investigations of AIE Behavior and pH-Sensitive Fluorescence Changes

Since TPE exhibited typical AIE characteristics, it was reasonable to deduce that the introduction of the TPE derivative would lead to AIE behavior of the polymer. The fluorescence spectra of the polymers were measured using a fluorescence spectrophotometer (F-2500 FL, Hitachi Ltd., Tokyo, Japan). In addition, the emission intensity of BT-PGA-TPE-HNPE in THF/water blends at various water fractions (f_w_) was observed [39]. To investigate whether BT-PGA-TPE-HNPE would change its fluorescence color in an acid environment, the fluorescence emission spectra of BT-PGA-TPE-HNPE at different pH were determined. The effect of incubation time on fluorescence change was also detected. The structural changes in the polymers at low pH were further analyzed by ^1^H NMR spectroscopy after taking equal amounts of the same samples and incubating them for 4 h at different pH conditions. The fluorescence quantum yield (ΦF) of BT-PGA-TPE-HNPE was calculated using quinine sulfate as the standard (ΦF = 55%) with the following formula [40].

φ_s_ =φ_r_ (A_r_·I_s_·η_s_^2^)/(A_s_·I_r_·η_r_^2^) (1)

where φ is the quantum yield; A, I and η denote the absorbance, the integration area of the emission spectrum and the refractive index of the solvent, respectively. The subscripts r and s denote the reference and the sample, respectively.

#### 3.4.3. Determination of Critical Micelle Concentration (CMC)

Due to the nature of AIE, the polymer could self-indicate the formation of micelles, providing a simple method for the determination of the CMC value. Specifically, a series of polymer in water dispersions with different concentrations (1 × 10^−4^–0.2 mg/mL) were prepared. The fluorescence (FL) intensity of samples at 546 nm was measured and the CMC value was calculated [41].

### 3.5. Preparation and Characterization of PTX-Loaded BT-PGA-TPE-HNPE Micelles

The PTX-loaded BT-PGA-TPE-HNPE micelles were prepared by a probe sonication method. Briefly, a certain amount of PTX in acetone solution (0.2 mL) was added to the BT-PGA-TPE-HNPE in a distilled water dispersion (10 mL). The combined solution was then subjected to ultrasonic agitation in an ice bath at 150 W for 5 min. Following this, it was dialyzed against distilled water using a membrane with a 7 k Da molecular weight cutoff for 2 h. After filtration through a 0.45 μm membrane, the resultant was lyophilized to obtain the PTX-loaded BT-PGA-TPE-HNPE micelles powder. Blank micelles were prepared without adding any PTX. The zeta potential and hydrodynamic diameter of the PTX-loaded micelles were analyzed using the dynamic light scattering (DLS) method (Zetasizer Nano-ZS90, Malvern Instruments, Malvern, UK). The presence form of PTX in the micelles was investigated by an X-ray diffraction (XRD) analysis (Geigerflex, Rigaku Co., Tokyo, Japan). The drug loading (DL) and encapsulation efficiency (EE) of the micelles were determined by a HPLC method [42].

DL (%) = weight of loaded drug/weight of drug-loaded micelles × 100%(2)

EE (%) = weight of loaded drug/weight of feeding drug × 100%(3)

### 3.6. In Vitro Drug Release Study

The in vitro drug release characteristics of the PTX-loaded micelles were investigated in PBS buffers with different pH values (pH of 7.4, 6.5, and 5.0). Typically, 4 mL of the PTX-loaded micelles were separately transferred into dialysis bags (MWCO of 7 k Da) and then immersed in 50 mL of PBS. Subsequently, incubation at 37 °C and 100 rpm was performed in an incubator shaker. At designed time intervals, 4 mL of external buffer was withdrawn and then replenished with equivalent fresh buffer. The amount of PTX released from the micelles was then determined by the HPLC method.

### 3.7. Cytotoxicity Investigation

The cytotoxicity of PTX-loaded BT-PGA-TPE-HNPE micelles was evaluated by a MTT assay. Briefly, 4T1 cells were inoculated into 96-well plates at 1 × 10^4^ cells/well and cultured for 24 h. When the cell confluence reached 80%, different concentrations of blank micelles, Taxol, or PTX-loaded micelles were added, and incubation proceeded at 37 °C for 24 h. Then 20 μL of MTT working solution (5 mg/mL) was added to each well, followed by a further incubation for 4 h. After that, 200 μL of DMSO was added. After shaking for 30 min, the absorbance of each well was measured at 490 nm by a microplate reader (Bio-Rad 680, Bio-Rad Laboratories, Hercules, CA, USA). The cell viability was calculated according to the following formula [43]:

Cell viability (%) = A_sample_/A_control_ × 100% (4)

where A_sample_ is the absorbance of the sample, and A_control_ is the absorbance of untreated cells.

To further investigate the cytotoxicity of drug-loaded micelles on tumor cells, a staining assay was performed using a Calcein acetoxymethyl ester/propidium iodide (Calcein-AM/PI) kit. In general, 4T1 cells were first treated with BT-PGA-TPE-HNPE micelles, Taxol, or PTX-loaded BT-PGA-TPE-HNPE micelles and then co-stained with Calcein AM and PI for 30 min. After washing twice with PBS solution, the cells were observed under a fluorescence microscope [44].

### 3.8. Cellular Imaging Capability and Cellular Uptake Behavior Investigation

The potential cell imaging ability of BT-PGA-TPE-HNPE micelles and their cellular uptake behavior were investigated using a laser confocal scanning microscope (CLSM, LSM 710, Zeiss, Oberkochen, Germany). 4T1 cells were inoculated into 6-well plates at 2 × 10^5^ cells/well. When the cell confluence reached 80%, the cells were incubated with BT-PGA-TPE-HNPE micelles or PGA-TPE-HNPE micelles for 0.5 h and 4 h. In addition, to investigate the effect of pH on the fluorescence behavior of BT-PGA-TPE-HNPE micelles, 4T1 cells were incubated with the micelles in PBS with different pH values (pH of 5.0, 6.5, and 7.4) for 4 h. The cell images were observed, and the fluorescence intensity was quantified by the Image J software.

### 3.9. In Vivo Antitumor Activity Study

The xenograft tumor model was established by subcutaneously injecting the 4T1 cell suspension into the right axilla of mice. When the tumor volume reached about 100 mm^3^, mice were randomly divided into three groups (*n* = 5). Normal saline, Taxol (PTX dose of 10 mg/kg), or PTX-loaded BT-PGA-TPE-HNPE micelles (PTX dose of 10 mg/kg) were injected 4 times through the tail vein at 3-day intervals. The tumor volume and the body weight of the mice were monitored every 2 days. At the end of the treatment, the tumors were excised, weighed, and photographed.

### 3.10. Statistical Analysis

Each experiment was performed at least three times, and all data were expressed as mean ± standard deviation. Statistical analysis was performed using one-way analysis of variance (ANOVA). A value of *p* < 0.05 was considered statically significant.

## 4. Conclusions

In summary, a novel visible polymeric micellar system was successfully fabricated. As expected, the BT-PGA-TPE-HNPE micelles were stable under physiological conditions and had excellent AIE properties and pH-sensitive discoloration. They emitted yellow fluorescence under normal physiological conditions but switched to blue fluorescence at the tumor site. Moreover, their high drug loading capacity and encapsulation efficiency significantly enhanced the antitumor efficacy. In vitro and in vivo experiments demonstrated the superior antitumor effect and negligible systemic toxicity of the micelles. The BT-PGA-TPE-HNPE micelles would be potential candidates for bioimaging and cancer therapy.

## Figures and Tables

**Table 1:** Comparison of BT-PGA-TPE-HNPE and CCBT micellar systems.

Items	BT-PGA-TPE-HNPE	CCBT
Quantum yield	26.6%	/
Synthesis steps	5	6
CMC	0.0186 mg/mL	0.021 mg/mL
Drug loading	15.0%	10.6%
Encapsulation efficiency	90.1%	83.4%
Cumulative drug release at a pH of 5.0	81.9%	<80%

## Data Availability

Data are contained within the article.
